#  Changing Trends in oral cancer - a global scenario 

**DOI:** 10.3126/nje.v6i4.17255

**Published:** 2016-12-31

**Authors:** Neha Gupta, Ritu Gupta, Arun Kumar Acharya, Basavaraj Patthi, Venkatesh Goud, Somanath Reddy, Anshul Garg, Ashish Singla

**Affiliations:** 1 Senior Lecturer, Department of Oral Medicine and Radiology, People's College of Dental Sciences and Research Bhopal, Madhya Pradesh- 462037; 2 Senior Lecturer, Department of Public Health Dentistry, D.J. College of Dental Sciences and Research, Modinagar, Uttar Pradesh, India - 201204.; 3 Professor and Head, Department of Public Health Dentistry, Navodaya Dental College and Hospital Raichur - 584102.; 4 Professor and Head, Department of Public Health Dentistry, D.J. College of Dental Sciences and Research, Modinagar, Uttar Pradesh, India – 2012014; 5 Senior Lecturer, Department of Public Health Dentistry, Malla Reddy Institute of Dental Sciences, Hyderabad, Telangana - 500055; 6 Senior Lecturer, Department of Public Health Dentistry, Navodaya Dental College and Hospital, Raichur - 584102; 7 Consultant and private practitioner, Department of ENT and Otolaryngology, Sanjeevani Clinic Ghaziabad, Uttar Pradesh - 201002; 8 Reader, Department of Public Health Dentistry, D.J. College of Dental Sciences and Research Modinagar, Uttar Pradesh, India - 2012014

**Keywords:** Cancer, Epidemiology, GLOBOCAN, Prevalence, Oral cavity

## Abstract

Oral cancer is one of the highly prevalent cancers worldwide and a leading cause of mortality in certain regions like South-Central Asia. It is a major public health problem. Late diagnosis, high mortality rates and morbidity are characteristics of the disease worldwide. For control of oral cancer an idea of the coverage of the same in the various regions is necessary. The estimated incidence, mortality and 5-year survival due to lip, oral cavity cancer in world is 3, 00, 373(2.1%), 1, 45, 328(1.8%) and 7, 02, 149(2.2%) respectively according to data of GLOBOCAN 2012. A changing trend in incidence and prevalence of oral cancer has been observed with more women and youngsters being affected by oral cancer.

## Introduction

Epidemiologic studies yield important information such as origin, prevalence and trends of certain diseases like oral cancer. Oral cancer is one of widely prevalent cancer types emerging as a growing problem in various regions of the world. Head and neck cancer is the 6th most common cancer in the world [ 1 ]. In South-central Asia, it is a common entity and the third most common type of cancer [ 2 ]. Squamous cell carcinomas are the most prevalent form of oral malignancies [ 1 ]. 

 Oral cancer is widely prevalent cancer type in developing countries and although it is less prevalent in developed western countries but in recent times a change in trend has been observed due to changes in lifestyle. It is the most common type of cancer in South Asian Countries like India, Srilanka, Pakistan and Bangladesh and contributes nearly one-fourth of all new cases of cancer [ 1, 3 ]. It is important to describe the epidemiological situation and coverage of oral cancer in various regions to facilitate the health policy makers in planning and implementing an effective prevention and control programme for oral cancer. Geographic location is an important variable for occurrence of oral cancer, as populations belonging to low socioeconomic status are at increased risk of developing oral cancer due to lack of awareness regarding ill-effects of preventable risk factors of oral cancer like tobacco and alcohol use. Epidemiology has a key role to play in the fight against cancer. Thus, the aim of this review is to highlight the recent data and study the trends in oral cancer globally.

### Method and Source of data

Global perspective on oral cancer is coordinated by the International Association of Cancer Registries (IARC), a component of World Health Organisation (WHO) via GLOBOCAN project. Data was extracted from the most recent GLOBOCAN 2012 database [ 4 ]. Also the data was compiled from relevant articles in the literature collected by National and International agencies.

### Regional Variations

A large variation in the distribution of oral cancer across various World Health Organisation (WHO) regions have been reported (Table 1) [ 4 ]. Oral cancer is characterised by marked geographical variations in its incidence and prevalence rates. South and Southeast Asia has high incidence rates for oral cancer with the exception of lip cancer. Countries like France and Hungary have reported high incidence of oral and pharyngeal cancer among men. Also high incidence is found in certain regions of Caribbean like Brazil and Puerto Rico, Pacific region like Melanesia and Papua New Guinea and in Western European region high incidence is reported in countries like Slovakia and Slovenia [ 1 ].

### Europe and European Union

In 2012, in Europe 73,860 and 25,770 of new cases of oral and pharyngeal cancers among males and females respectively have been reported accounting to a total of 99,630 new cases. Similarly in 2012 in European Union (EU) 53,370 and 19,650 among males and females respectively have been reported [ 5 ]. A high incidence of oral and pharyngeal cancer among males were reported in Russian Federation and Germany whereas countries like Cyprus and Iceland have reported low incidences. Also, though Central and Eastern European regions have lower reported incidence rates compared to Western European regions, they have high mortality rates due to oral and pharyngeal cancers [ 1 ]. The highest estimated age-standardised rates (ASRs) among males have been reported in France and Hungary and among females in Denmark and Hungary [ 5 ]. 

 The age-standardised incidence rate (ASIR) across all geographic regions of United Kingdom (UK) for which estimates are available is 4.6 per 100,000 population. An estimated 1,296 deaths with age standardised mortality rate (ASMR) of 1.0 has been reported in 2012 in United Kingdom [ 4 ].

### North America

In United States of America (USA), cancers of the oral cavity and oropharynx constitutes 3% of all the malignancies in men and 2% in women [ 6 ]. The estimated number of new cases in US, 2013 is 41,380 for oral cavity and pharynx cancer, 13,590 for tongue cancer and 11,400 for mouth cancer [ 7 ]. In 2012, an estimated 26,064 (1.6%) new cases were diagnosed of lip and oral cavity cancer. The ASIR across all geographic regions of United States of America for which estimates are available is 5.2 per 100,000 population. An estimated 4,620 deaths have occurred in 2012 due to cancer of lip and oral cavity, with the ASMR being 0.8. It is the 11th most common cancer in USA among males while in Canada and Mexico it is the 12th and 13th most common cancer respectively. The ASIR for lip and oral cavity cancer among men in Canada and Mexico is 4.2 and 3.1 respectively [ 4 ].

### South America

In South America, for cancer of the lip and oral cavity, 15,868 (2.0%) cases have been estimated to be diagnosed in 2012. The age-standardised incidence rate across all geographic regions of South America for which estimates are available is 3.8 per 100,000 population. An estimated 6,046 deaths have occurred in 2012 in South America due to lip and oral cavity cancer, with the age-standardised mortality rate being 1.4 [ 4 ]. 

 In Brazil, lip and oral cavity cancer is the 7th most common cancer, with an estimated 6,930 new cases diagnosed in the year 2012. The ASIR across Brazil for which estimates are available is 7.2 per 100,000 population and an estimated 3,020 deaths have occurred due to lip and oral cavity cancer [ 4 ].

### Africa

There is paucity of quality data in Africa and only data from few hospital based cancer registries is available [ 1 ]. 

 According to GLOBOCAN 2012, lip and oral cavity cancer is the 15th most common cancer in Africa and 7th most common cancer in Middle Africa. In 2012, an estimated 17,276 new cases of lip and oral cavity cancer were diagnosed. The standard incidence rate in Africa is 2.6 per 100,000 population, ranging from 1.5 in Western Africa to 4.0 in Southern Africa. A total of 10,341 deaths have occurred in Africa due to lip and oral cavity cancer and the age-standardised mortality rate is 1.6, with highest mortality rate being reported in Eastern Africa (2.2%) and Middle Africa (2.3%) [ 4 ].

### Asia

According to GLOBOCAN 2012, lip and oral cavity cancer is the 12th most common cancer in Asia and ranks 8th among all the cancers in men, with an estimated 16,88,50 new cases of lip and oral cavity cancer diagnosed in the year 2012. The standard incidence rate in Asia is 3.8. An estimated 97,408 deaths have occurred in Asia due to lip and oral cavity cancer and the age-standardised mortality rate is 2.2. It is the second most common cancer among men in South-Central Asia, with an age-standardised incidence of 9.9 and a 5-year prevalence of 129,057 (12.1%). The Eastern and Western parts of Asia have low incidence rate of cancer of lip and oral cavity with a standard incidence rate of 1.8 and 2.1 respectively [ 4 ]. 

 Oral cancer is highly prevalent in South Asian countries like Bangladesh, India, Pakistan and Sri Lanka, where one-third of all the cancers reported are oral cancer. Majority (90%) of the cases reported of oral cancer is attributed to tobacco consumption in various forms in these regions [ 8 ]. According to GLOBOCAN 2012 data, Sri Lanka (10.3) has the highest ASR of incidence in South Asia and the cancer of the lip and oral cavity is the most common cancer among men in Srilanka [ 4 ]. Pakistan has the second highest ASIR of lip and oral cavity cancer (9.3) and is the most common cancer among men in Pakistan [ 4 ]. In China, a study by Zhang SK et al; reported the crude incidence rate of oral cancer

### Oceania

According to GLOBOCAN 2012, lip and oral cavity cancer is the 12th most common cancer in Oceania, with an estimated 3,631 new cases of lip and oral cavity cancer diagnosed in the year 2012. The age-standardised incidence rate in Oceania is 7.4. An estimated 1,145 deaths have occurred in Oceania due to lip and oral cavity cancer and the age-standardised mortality rate is 1.9 [ 4 ]. 

 In Australia, cancer of lip and oral cavity is the ninth most common cancer among men with standard incidence rate of 8.8, while in New Zealand it is 14th most common cancer among men with ASRs of incidence being 5.5 [ 4 ]. Papua New Guinea and Soloman Islands have reported high prevalence of oral cancer largely attributable to tobacco smoking and betel quid chewing, whereas lower rates were found in Vanuatu [ 10 ].

## Descriptive patterns

### According to site

Among European and US populations tongue is the most common site accounting to nearly half of all oral cancers reported. High rates of tongue cancer are also reported in Brazil and Sao Paulo. But in Asian countries like India, buccal mucosa is the most common site of oral cancer as reported in Maharashtra, India [ 11 ] and in Khon Kaen, Thailand [ 12 ] whereas a study in Allahabad, India reported tongue as the most common site of potentially malignant and malignant oral lesions [ 13 ]. Also a review conducted by Rao SVR et al, found tongue as the commonly affected site in oral cancers in India and other common sites affected are buccal mucosa and gingiva in Asian countries [ 14 ].

### Gender

The gender differences in some population groups could be attributed to the variations in habits of tobacco consumption and lifestyle. Oral cancer is more prevalent among men because of heavy tobacco and alcohol consumption among them. But in recent times women are also being greatly affected by oral cancer due to changes in lifestyle. A study conducted to estimate gender differences and risk for oral cancer, found women with smoking habits had higher risk of developing oral cancer compared to male smokers. There was increased risk among women for developing oral cancer with the habit of smoking and alcohol intake when compared to men. Also hormonal changes, nutritional deficiencies of iron, riboflavin, vitamins and minerals increase the risk of oral cancer among older women [ 15 ]. A study in Thailand found standard incidence rate of oral cancer to be significantly higher in women compared to men [ 12 ].

### Ethnic Variations

Prevalence of oral cancer is influenced by ethnicity due to strong influence of social and cultural practices [ 1 ]. Cultural practices are important risk factors for development of oral cancer. Immigration from regions with high incidence of cancer to other regions of the world results in higher incidence in immigrant communities. Migrant studies relating to oral cancer are meagre [ 8 ]. 

 In South Africa, a study on ethnic disparities in oral and oro-pharyngeal cancers, found high ASIR among females of Asian/Indian South Africans. It was also high among Coloureds and lowest among Blacks [ 16 ]. In USA, study on various ethnic groups found increasing rate of cancer of tongue among young white female adults and was also higher among females in Blacks, Hispanics and Asians [ 17 ]. But a study in UK found a weak correlation between ethnicity and oral cancer in UK population [ 18 ].

### Survival and Mortality

According to the World Health Organization (WHO) 10.3 million people might die due to cancer by the year 2020 [ 19 ]. 

 Globally a low 5-year survival rate of 50% is seen among patients with oral cancer [ 20 ]. Despite oral cavity being accessible for examination, individuals report to a clinician only in later stages of malignancy, thus no improvement in survival rate for oral cancer over the decades have been observed [ 6 ]. 

 Within United States, there is a greater than five-fold regional variation, state by state, in mortality rates [ 8 ]. These regional differences are explicable in terms of a combination of ethnic mix, socioeconomic mix and variations in the prevalence and severity of risk factors in these communities, including the use of oral smokeless tobacco in the southern states. Aside from these important regional and ethnic differences, the overall downward trend in the USA is encouraging [ 8 ]. 

 Even more striking country-to-country variations are seen within the EU, where the number of deaths due to oral cancer has doubled between 1970 and 2000. Between 3-fold and 10-fold increase in single generation in Central and Eastern Europe for oral cancer has been observed [ 8 ]. 

 Much information is now available to indicate a rise in both incidence and mortality due to oral cancer in many other parts of the world. The highest increases are in countries of Central, and then of Western Europe, including parts of Spain. No improvement in the survival rate has been observed since last three decades in the United Kingdom [ 1 ]. Among the Asian countries, South Central Asia has the highest reported age standardised mortality rate of 3.0. The prognosis of oral cancer decreases as the disease advances. Women are found to have higher survival rates in cancer of tongue and oral cavity when compared to men [ 1 ].

### Changing Trends

Cohort studies show that oral cancer incidence has risen in all the age groups throughout the world in the last decade, especially in young men in Eastern European countries, such as, USA, Estonia, Hungary, Ukraine, and Russia [ 17, 21, 2 ]. 

 In the recent times, European countries like Hungary have reported two-fold increase in incidence and mortality due to oral cancer. Also an average 2.7% increase in the age standardised incidence of oral cancer has been observed in United Kingdom since 1989 [ 1 ]. 

 Consumption of tobacco in its various forms is found to be the leading cause of cancer in regions with high prevalence rates and majority of the patients are below 35-years of age [ 23 ]. 

 A change in trend for oral cancer was observed in USA between 1995-2004 with a significant decrease in incidence among men with an annual percentage change (APC) of 1.5% for all races (1.6% for men and 1.8% for women). Similarly in France between 1978 and 2000 a decrease in incidence of cancer of oral cavity and pharynx among men with APC of 1% and an increase in incidence among women with APC of +1.73 were observed [ 1 ]. Males to females ratio with cancer of mouth has declined over the years, also a decrease in the prevalence of lip cancer has been observed [ 1,  23 ].

### Increase in Youngsters

Although there has been a declining trend in the overall incidence of oral cavity squamous cell carcinoma over the past 30 years, recent studies suggest the incidence of this disease in young adults may be on the rise worldwide. Prevalence of 11.3% of oral cancer is reported among patients below 45 years of age according to Surveillance, Epidemiology and End Results (SEER) data [ 24 ]. Also increased incidence and mortality rate among young adults below 40 years in EU and USA have been reported [ 25, 6 ].

## Conclusion:

 Oral cancer is largely caused due to preventable factors like tobacco consumption. The increase in life expectancy and changes in lifestyle have all contributed to the increase in oral cancer rates in the world. High prevalence of oral cancer is found worldwide with highest prevalence rate reported in South-Central Asia. Epidemiological data should be used to determine an appropriate and optimal integrated cancer control programme with existing health care services and prevention strategies to decrease the growing threat of oral cancer worldwide. Conflict of interest: None .

## Figures and Tables

**Table 1:** Table 1. Estimated incidence, mortality and 5-year prevalence of lip, oral cavity cancer. Source: GLOBOCAN 2012 (IARC) [4]Incidence and mortality data for all ages. 5-year prevalence for adult population only. ASR (W) and proportions per 100,000. N – Number ASR (W) – Age-standardised rate WHO – World Health Organisation Prop -Proportion

**Region**		**Incidence**		**Mortality**		**5-year prevalence**	
		**N (%)**	**ASR (W)**	**N (%)**	**ASR (W)**	**N (%)**	**Prop**
**World**	Men	198975 (2.7)	5.5	97940 (2.1)	2.7	467157 (3.1)	18.0
	Women	101398 (1.5)	2.5	47413 (1.3)	1.2	234992 (1.4)	9.1
	Both sexes	300373 (2.1)	4.0	145353(1.8)	1.9	702149 (2.2)	13.5
**WHO African Region**	Men	80509 (3.0)	3.4	5026 (2.4)	2.2	18446 (3.9)	7.3
	Women	5475 (1.4)	2.0	3504 (1.4)	1.4	12766 (1.4)	4.9
	Both sexes	13484 (2.1)	2.7	8530 (1.9)	1.8	31212 (2.3)	6.1
**WHO American Region**	Men	31898 (2.2)	5.9	8532 (1.3)	1.5	94953 (2.5)	26.9
	Women	17302 (1.2)	2.6	4271 (0.7)	0.6	48526 (1.2)	13.1
	Both sexes	49200 (1.7)	4.2	12803 (1.0)	1.0	143479 (1.8)	19.9
**WHO East Mediterranean Region**	Men	11601 (4.4)	5.1	6185 (3.2)	2.8	27236 (5.9)	12.9
	Women	9080 (3.1)	4.1	4812 (2.7)	2.3	21570 (2.9)	10.7
	Both sexes	20681 (3.7)	4.6	10997 (3.0)	2.5	48806 (4.1)	11.8
**WHO Europe Region**	Men	45567 (2.3)	7.1	18642 (1.7)	2.9	118151 (2.5)	33.2
	Women	20366 (1.2)	2.4	6560 (0.8)	0.7	51933 (1.1)	13.4
	Both sexes	65933 (1.8)	4.6	25202 (1.3)	1.7	170084 (1.8)	22.8
**WHO South East Asian Region**	Men	70816 (8.7)	8.9	45247 (7.3)	5.7	122976 (9.9)	18.4
	Women	32648 (3.6)	4.0	20487 (3.7)	2.5	58034 (2.8)	8.9
	Both sexes	103464 (6.0)	6.4	65734 (5.6)	4.1	181010 (5.5)	13.7
**WHO Western Pacific Region**	Men	31013 (1.2)	2.7	14292 (0.8)	1.2	85233 (1.9)	11.4
	Women	16511 (0.9)	1.3	7776 (0.7)	0.6	42123 (0.9)	5.8
	Both sexes	47524 (1.0)	2.0	22068 (0.7)	0.9	127356 (1.4)	8.6
